# Case report: Concurrent MOG antibody-associated disease and latent infections in two patients

**DOI:** 10.3389/fimmu.2024.1455355

**Published:** 2024-09-04

**Authors:** Laila Kulsvehagen, Tim Woelfle, Ana Beatriz Ayroza Galvão Ribeiro Gomes, Patrick Lipps, Tradite Neziraj, Julia Flammer, Karoline Leuzinger, Tobias Derfuss, Jens Kuhle, Athina Papadopoulou, Anne-Katrin Pröbstel

**Affiliations:** ^1^ Department of Neurology and Research Center for Clinical Neuroimmunology and Neuroscience Basel (RC2NB), University Hospital Basel and University of Basel, Basel, Switzerland; ^2^ Departments of Biomedicine and Clinical Research, University Hospital Basel and University of Basel, Basel, Switzerland; ^3^ Translational Imaging in Neurology (ThINk) Basel, Department of Biomedical Engineering, University Hospital Basel and University of Basel, Basel, Switzerland; ^4^ Departamento de Neurologia, Instituto Central, Hospital das Clínicas, Faculdade de Medicina, Universidade de Sao Paulo (HCFMUSP), Sao Paulo, Brazil; ^5^ Clinical Virology, University Hospital Basel, Basel, Switzerland

**Keywords:** myelin oligodendrocyte glycoprotein, myelin oligodendrocyte glycoprotein antibody-associated disease, syphilis, infection, case report, molecular mimicry, antibodies, B cells

## Abstract

**Objectives:**

Myelin oligodendrocyte glycoprotein (MOG) antibody-associated disease (MOGAD) is frequently preceded by infections. The underlying pathomechanism, however, remains poorly understood. Here, we present the clinical data of two MOGAD patients with concurrent syphilis infection and investigate the reactivity of patient-derived antibodies to MOG and *Treponema pallidum* (*T. pallidum*).

**Methods:**

Longitudinal serum samples and soluble immunoglobulins in single B cell supernatants were measured for MOG reactivity by a live cell-based assay. Reactivity against *T. pallidum* was assessed by enzyme-linked immunosorbent assay.

**Results:**

The two patients presented MOGAD and concurrent latent syphilis infection, manifesting as cervical myelitis and unilateral optic neuritis, respectively. The first patient had been living with HIV on antiretroviral therapy, and the second was concomitantly diagnosed with chronic hepatitis B infection. Upon screening of B cell supernatants, we identified reactivity to MOG or *T. pallidum.* Notably, one B cell showed reactivity to both antigens.

**Discussion:**

The coexistence of MOGAD diagnoses and latent syphilis, alongside the identification of antibody reactivity to MOG and *T. pallidum*, underscores the potential pathomechanistic link between syphilis infection and subsequent autoimmune neuroinflammation. Cross-reactivity between MOG and *T. pallidum* antibodies remains to be validated on a molecular level, and further characterization of infectious triggers associated with MOGAD is needed.

## Introduction

Myelin oligodendrocyte glycoprotein (MOG) antibody-associated disease (MOGAD) is a recently classified autoimmune demyelinating disease entity of the central nervous system (CNS), characterized by the presence of specific core clinical features and antibodies against MOG (1, 2). Importantly, MOGAD is frequently preceded by infections or vaccinations and often follows a monophasic disease course (1), suggesting a parainfectious etiology. Potential mechanisms include molecular mimicry, epitope spreading, and bystander activation of autoreactive lymphocytes. Yet, molecular evidence linking infection to the generation of a MOG-specific B cell response is missing.

## Methods

Imaging and (para)clinical data were retrieved from hospital records. Available serum samples were measured for MOG-IgG by a live cell-based assay (CBA), as previously described (3). In brief, sera (1:100) and cerebrospinal fluid (CSF) (1:5) were examined for IgG reactivity against full-length human MOG using a live cell-based assay. For each sample, the ratio of the geometric mean channel fluorescence intensity (geometric MFI) of the MOG-transfected cell line divided by the geometric MFI of the control cell line was calculated. Values below 2.4 are considered negative, values between 2.4 and 3 positive, and values above 3 clear-positive.

To investigate the potential cross-reactivity of patient-derived B cells, B cells reactive to MOG were enriched from the two patients with an established protocol (4). In brief, B cells were isolated from peripheral blood mononuclear cells by magnetic negative selection and cultivated with a stably transfected cell line to allow antigen uptake. Here, we used a cell line expressing full-length human MOG fused to a green fluorescence protein to allow flow cytometry cell sorting of MOG-recognizing B cells. Supernatants of single-plated B cells were screened for immunoglobulin reactivity against MOG by CBA, and reactivity against common *T. pallidum* antigens (TpN15+TpN17+TpN47, Alpha Diagnostic, San Antonio, USA) was measured by enzyme-linked immunosorbent assay. Both patients provided written informed consent, and CARE reporting guidelines were followed.

## Results

We present two cases of patients with diagnoses of latent syphilis and MOGAD with clear-positive MOG-IgG levels according to the International MOGAD Panel proposed criteria (2), hereby expanding on the existing literature to a total of five reported cases of concurrent syphilis infection and MOGAD (**Table 1**).

**Table 1:** Case summaries of concurrent MOGAD and syphilis infection.

Publication	Age (y), sex	Neurological diagnosis	MOG-Ig positivity at onset; serum, CSF [titer or MFI ratio]	Syphilis stage	Co-infections
**Gudenkauf et al., *Neuroimmunology Reports.* 2021 (** 13)	31, male	Meningoencephalomyelitis	Positive [1:80], N/A	Latent	None
**Jeyakumar et al., *Neuroimmunology Reports.* 2021 (** 14)	45, male	Papillitis, intermediate uveitis	N/A^a^, positive	Neurosyphilis	None
**Shi et al., *Front Neurol.* 2023 (** 15)	37, male	Optic neuritis	Positive [1:200], positive [1:200]	Neurosyphilis	None
**Kulsvehagen et al., *Front Immunol.* 2024**	43, male	Cervical myelitis	Positive [14], N/A	Latent	HIV, latent tuberculosis
**Kulsvehagen et al., *Front Immunol.* 2024**	44, male	Optic neuritis	Positive [20], borderline positive [3]	Latent	Hepatitis B

Summary of five reported patients with coexisting (neuro)syphilis and MOGAD. MOG-IgG titers and MFI ratios were included when available.

CSF, cerebrospinal fluid; MOG, myelin oligodendrocyte glycoprotein; N/A, not available; HIV, human immunodeficiency virus.

a The serum sample at disease onset was not available (the serum sample collected 2 months after disease onset was MOG-IgG negative).

### Case 1

A 43-year-old man was admitted to our service with a 2-day history of numbness in the genital area and lower limbs. He had been living with human immunodeficiency virus (HIV) for 5 years and was on antiretroviral therapy with elvitegravir, cobicistat, emtricitabine, and tenofovir. The lowest initial CD4 count was 406/µl (35.8%) and the highest viral load was 304,000 cp/ml. The patient’s history suggested that primary HIV infection occurred 3 months before diagnosis and treatment was initiated right away, leading to sustained viral suppression. Additionally, he reported previous diagnoses of untreated latent tuberculosis 1 year prior and latent syphilis 2 years prior, which was treated with benzathine penicillin. One year before neurological symptom onset, a lab test confirmed negative serology for syphilis as assessed by the venereal disease research laboratory (VDRL) test, indicating adequate treatment response.

**Figure 1:**
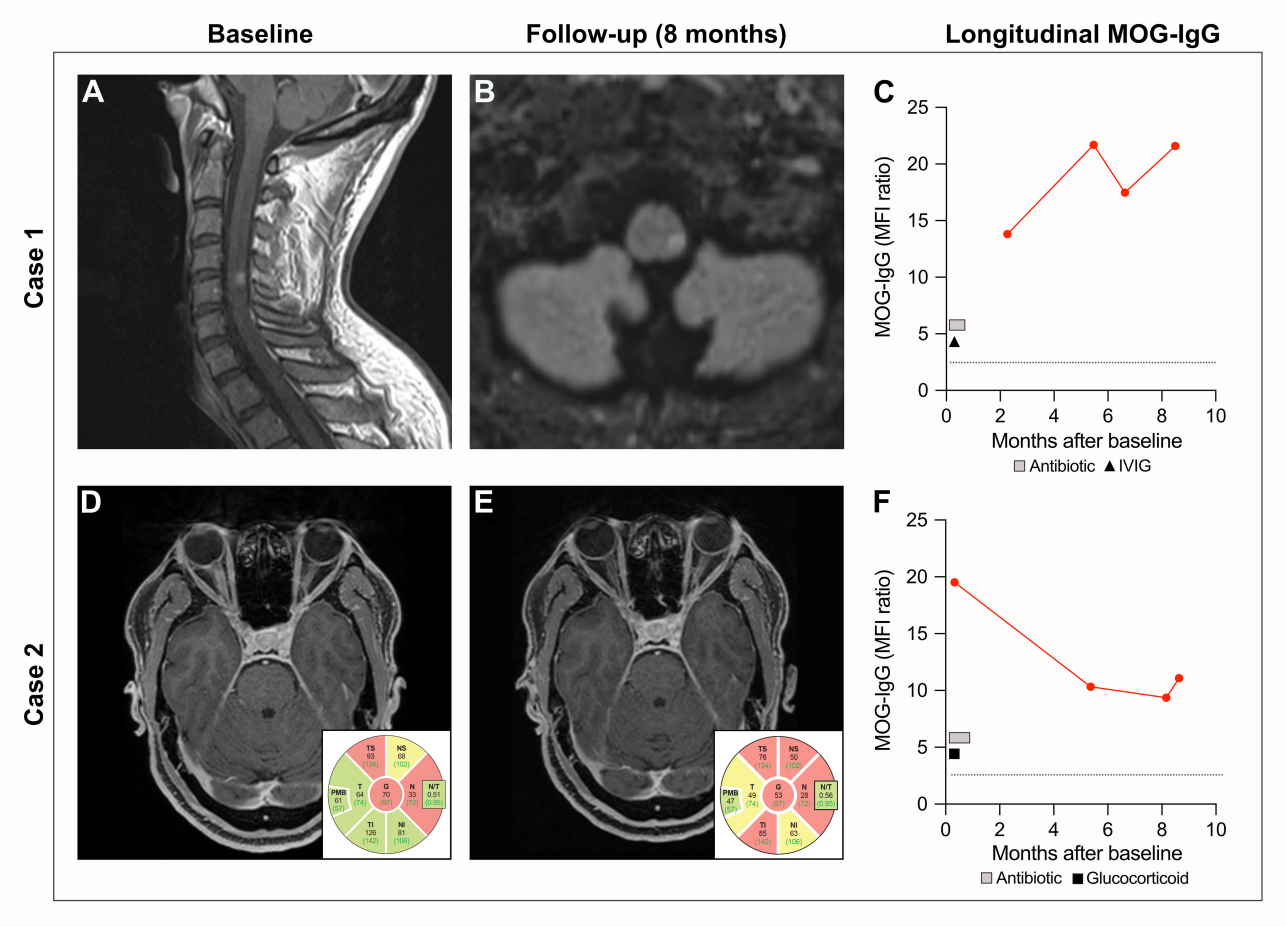
Clinical features at baseline and follow-up. Case 1: **(A)** Spinal MRI with sagittal T1 showing contrast-enhancing lesion on the level of cervical vertebrae 4/5, presenting as numbness in the genital area and both legs. **(B)** Cerebral MRI with transversal FLAIR revealing a new lesion in the left brainstem 6 months after initial treatment with IVIG. **(C)** Longitudinal MOG-IgG values (geometric MFI ratio). The dotted line indicates the cutoff for clear-positive MOG-IgG signals (geometric MFI ratio = 3). The square indicates treatment with an antibiotic (intravenous penicillin [20 million units/day] for 14 days) and the triangle indicates IVIG treatment (150 g over 5 days). Case 2: **(D)** Transversal T1 showing contrast enhancement of the right optic nerve at diagnosis. OCT showing slightly (yellow) and severely (red) diminished pRNFL thickness of the right eye. **(E)** Transversal post-contrast T1 showing bilateral normal optic nerves; however, OCT showed a decrease of the pRNFL thickness of the right eye 8 months after initial treatment with methylprednisolone. **(F)** MOG-IgG values (geometric MFI ratio) at baseline and follow-up time points. Cutoff for clear-positive MOG-IgG signals (geometric MFI ratio = 3) indicated as a dotted line. The square indicates treatment with an antibiotic (benzathine penicillin [2.4 million U/week] over 3 weeks) and the triangle indicates treatment with glucocorticoids (methylprednisolone 3 g over 3 days). MRI, magnetic resonance imaging; FLAIR, fluid-attenuated inversion recovery; IVIG, intravenous immunoglobulin; MOG, myelin oligodendrocyte glycoprotein; MFI, mean fluorescence intensity; OCT, optical coherence tomography; pRNFL, peripapillary retinal nerve fiber layer.

On full neurological examination, he presented lower limb hypoesthesia with a sensory level at T12, not associated with hypalgesia, pallhypesthesia, thermhypesthesia, motor, or autonomic dysfunction; reflexes were symmetrical. Magnetic resonance imaging (MRI) of the spinal cord revealed a gadolinium-enhancing T2-signal hyperintense lesion at the level of C4 and C5 (**Figure 1**). Serologies for syphilis with the VDRL test presented a titer of 1:16, which had previously been negative for over a year, and an increased *T. pallidum* particle agglutination (TPPA) titer of 1:20,480 after being stable at 1:640 for more than a year. CSF was normal, with no signs of pleocytosis, disturbed blood–CSF barrier, or intrathecal immunoglobulin production, except for a TPPA of 1:16. VDRL was also negative. The patient was diagnosed with latent syphilis reinfection or reactivation in association with short cervical myelitis. Due to the near-normal CSF analysis and atypical clinical presentation, diagnosis of neurosyphilis was considered unlikely. Additional opportunistic infections were ruled out at neurological presentation: VZV IgG was positive in serum but negative in CSF, and IgM was negative in both serum and CSF. CMV IgG had been positive prior to neurological presentation and remained unchanged. HSV 1 + 2 IgG was positive in serum and negative in CSF, and IgM was borderline positive in serum (most likely unspecific) and negative in CSF. *Borrelia* and FSME IgG and IgM were negative in both CSF and serum. EBV and HIV PCR were negative in CSF.

The patient was treated with intravenous penicillin (20 million units/day) for 14 days and 150 g of intravenous immunoglobulin (IVIG) over 5 days. He responded well to treatment and presented a fast partial recovery with the presence of a residual Lhermitte’s sign. A MOG-IgG test in serum sampled at the 2-month follow-up was clear-positive (2) (**Figure 1**, **Table 2**). At the 8-month follow-up, the patient presented new asymptomatic pontine and medullary lesions on the MRI (**Figure 1**). The clear-positive MOG-IgG serum levels increased over time, while neurofilament light-chain (sNfL) levels remained within the same range (**Table 2**). The patient was diagnosed with MOGAD. While there may be a certain risk of a false-positive serum MOG-IgG in the 2-month measurement due to IVIG treatment, this cannot explain the positive results obtained at follow-up, especially at 9 months. The risk of attack recurrence was discussed with the patient, and we recommended maintenance treatment with intravenous immunoglobulin. While the patient was satisfied with the initial treatment response, he was opposed to chronic treatment and preferred a wait-and-see approach.

**Table 2:** Demographic and (para)clinical features.

	Case 1	Case 2
**Age (y), sex**	43, male	44, male
**Initial symptoms**	Numbness in the genital area and lower limbs	Vision loss and pain in the right eye
**Neurological diagnosis**	Cervical myelitis	Optic neuritis
**Syphilis serology**	TPPA+ (1:20,480), VDRL+ (1:16)	TPPA+ (1:1,280), VDRL−
**Serum MOG-IgG (months after disease onset)**	Month 2: geometric MFI ratio of 14 Month 5: geometric MFI ratio of 22 Month 7: geometric MFI ratio of 17 Month 9: geometric MFI ratio of 22	Month 0: geometric MFI ratio of 20 Month 5: geometric MFI ratio of 10 Month 8: geometric MFI ratio of 9 Month 9: geometric MFI ratio of 11
**Serum NfL (months after disease onset)**	Month 2: 5.4 pg/ml (15 percentile) Month 5: 4.0 pg/ml (2.3 percentile) Month 7: 5.2 pg/ml (13 percentile) Month 9: 5.1 pg/ml (12 percentile)	Month 0: 10.7 pg/ml (88 percentile) Month 5: 8.8 pg/ml (71 percentile) Month 8: 10.2 pg/ml (84 percentile) Month 9: 14.9 pg/ml (97 percentile)
**Lumbar puncture**	2 leukocytes, protein 440mg, OCB negative, TPPA positive (1:16), VDRL negative	2 leukocytes, protein 340 mg, OCB negative, TPPA positive (1:2), VDRL negative
**Anti-AQP4**	Negative	Negative
**Neurological treatment**	IVIG 150 g over 5 days	Methylprednisolone 3 g over 3 days
**Syphilis treatment**	Penicillin 20 million U per day over 14 days	Benzathine penicillin 2.4 million U per week over 3 weeks
**Comorbidities**	HIV CDC A2 (bictegravir, emtricitabine, tenofovir), latent tuberculosis (untreated)	Hepatitis B (tenofovir)
**Follow-up (8 months)**	Asymptomatic new FLAIR lesions in the pons and medulla oblongata and increase in MOG-IgG serum level	Decrease in pRNFL thickness (as expected after optic neuritis) and persistent MOG-IgG seropositivity

For serum MOG-IgG levels, values below 2.4 are considered negative, values between 2.4 and 3 positive, and values above 3 clear-positive.

TPPA, Treponema pallidum particle agglutination; VDRL, venereal disease research laboratory; OCB, oligoclonal band; IVIG, intravenous immunoglobulin; HIV, human immunodeficiency virus; CDC, Centers for Disease Control and Prevention; FLAIR, fluid-attenuated inversion recovery; MOG, myelin oligodendrocyte glycoprotein; MFI, mean fluorescence intensity; NfL, neurofilament light chain.

### Case 2

A 44-year-old man was referred to our service with a 1-week history of vision loss and pain in the right eye. Ophthalmological examination revealed a right eye visual acuity of 10/50 with red color desaturation (Ishihara 5/13 on the right eye, 13/13 on the left eye), and full neurological examination was normal. The brain MRI showed optic disk edema and a gadolinium-enhancing T2-signal hyperintensity of the intraorbital and intracanalicular segment of the right optic nerve, nerve sheath, and adjacent fatty tissue suggesting a diagnosis of optic neuritis (ON) (**Figure 1**). Serological investigation revealed chronic hepatitis B virus (HBV) infection and suspected latent syphilis infection over neurosyphilis based on VDRL being negative (TPPA 1:1,280, VDRL negative). The CSF analysis was normal, except for a TPPA of 1:2. The patient was treated with intravenous methylprednisolone (1 g/day for 3 days), tenofovir (245 mg/day), and intramuscular benzathine penicillin (2.4 million units/week over 3 weeks). He responded with full visual acuity recovery and minor color desaturation after 1 week. At follow-up, a clear-positive (2) MOG-IgG serum result sampled at hospital admission was detected, leading to the diagnosis of MOGAD (**Figure 1**, **Table 2**). At the 8-month follow-up, no new lesions were detected on the MRI. Yet, sNfL and MOG-IgG levels remained high (**Figure 1**, **Table 2**). Optical coherence tomography (OCT) showed further decrease of the peripapillary retinal nerve fiber layer thickness (pRNFL) in the right eye (**Figure 1**). The patient was diagnosed with monophasic MOGAD, but regular follow-up examinations were appointed. The patient was satisfied with the treatment response and regular follow-ups.

### Experimental results

**Figure 2:**
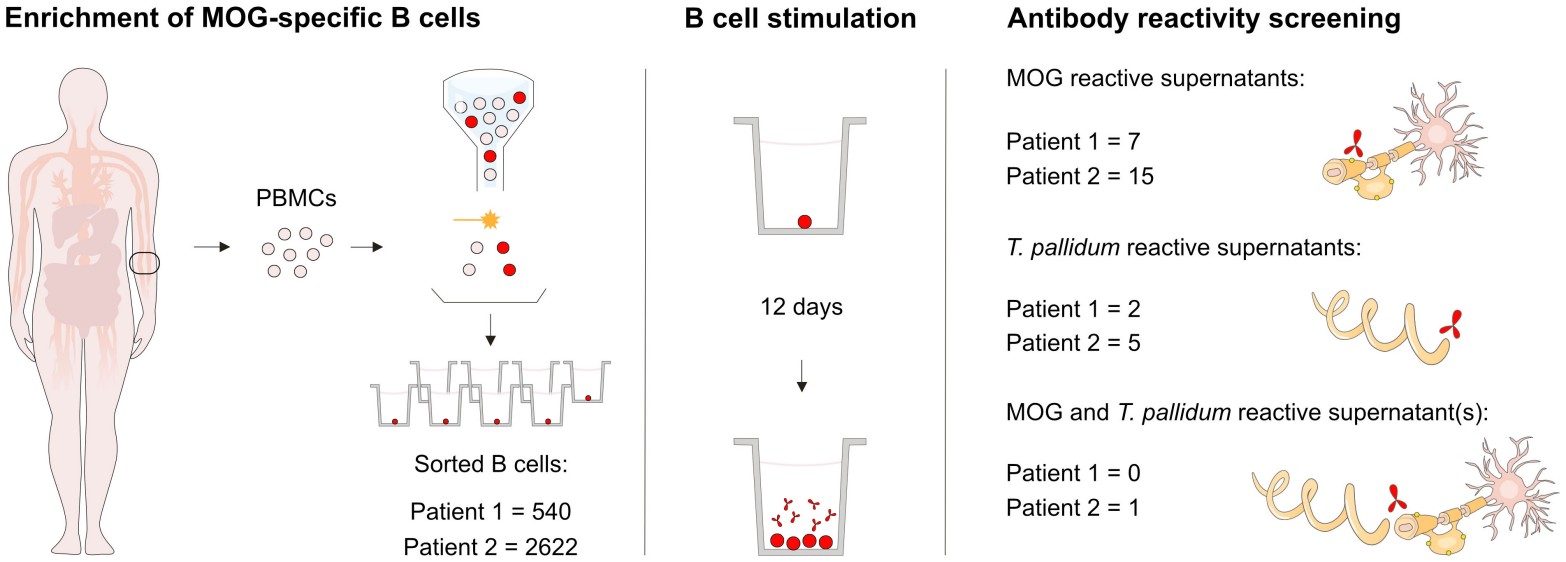
Experimental design. Schematic illustration of the experimental setup and the number of isolated B cell supernatants with detected reactivities to MOG and *T. pallidum*. MOG, myelin oligodendrocyte glycoprotein; *T. pallidum*, *Treponema pallidum*; PBMCs, peripheral blood mononuclear cells.

To examine whether the patients had B cells reactive to MOG and *T. pallidum*, we assessed the reactivity of isolated B cell supernatants from the two patients *in vitro*. For the first patient, from 19 million peripheral blood mononuclear cells (PBMCs), we obtained 1.3 million cells after B cell enrichment, of which 540 were gated as MOG-recognizing B cells. These were sorted and single-plated, of which a total of seven supernatants recognized MOG and two showed reactivity to *T. pallidum* antigens (**Figure 2**). From the second patient, from 12 million PBMCs, we obtained 1.3 million cells after B cell enrichment, and 2,622 enriched B cells were sorted and single-plated. Of these, reactivity to MOG was identified in 15 supernatants, whereas reactivity to *T. pallidum* antigens was detected in five supernatants (**Figure 2**). Notably, of these supernatants, one showed reactivity to both MOG and *T. pallidum.* Measurements of positive B cell supernatants were repeated and validated in independent experiments.

## Discussion

The current study expands on the existing literature of concurrent infections in MOGAD by reporting two MOGAD cases with latent syphilis infection among other latent infections, specifically HIV, tuberculosis, and HBV, respectively. Previously reported infections associated with MOGAD include respiratory infections (2, 5) (e.g., SARS-CoV-2) (6), Zika virus (7), mumps (8), herpes simplex virus (9), *Borrelia* (10), Epstein–Barr virus (11), and tuberculosis (12). Furthermore, three cases of MOGAD and coexisting syphilis infection have been described: one patient with latent syphilis and meningoencephalomyelitis associated with MOG-IgG seropositivity (13), one patient with acute neurosyphilis (presenting with papillitis and uveitis) and positive MOG-IgG results in the CSF (14), and one case of coexisting neurosyphilis and MOGAD manifesting as ON (15), a rare manifestation of neurosyphilis that shows similarities to ON in MOGAD (16). Due to infections being commonly reported in MOGAD, the hypothesis of post-infectious generation of MOG-specific antibodies has been suggested (1). The proposed underlying mechanisms include molecular mimicry, epitope spreading, bystander activation, and polyclonal B cell activation. Yet, molecular evidence linking specific infectious triggers to the generation of autoreactive MOG-specific B cells is missing.

The identification of a patient-derived B cell showing reactivity to both MOG and *T. pallidum* in one of the patients with MOGAD and concurrent syphilis infection suggests a potential link between this infectious antigen and the development of autoreactive B cells through molecular mimicry. However, whether cross-reactivity is indeed involved in the pathomechanism of the autoimmune responses in MOGAD remains to be validated on a molecular level. For instance, this may be assessed by investigating the cross-reactivity of MOG-specific patient-derived monoclonal antibodies. Furthermore, although we identified a B cell reactive to MOG and *T. pallidum*, we cannot rule out the involvement of additional coexisting infections in these patients. In fact, cross-reactivity between HBV surface antigen and MOG peptides has been reported previously (17), and it has been suggested that syphilis infection can lead to Epstein–Barr virus reactivation and hepatitis (18). To further characterize the link between infectious triggers and MOGAD, additional molecular and functional studies investigating cross-reactivity to antigens from additional infections are warranted. Moreover, to prevent diagnostic delay and ensure optimal treatment strategies, testing of MOG-Ig in serum, including MOG-IgA as recently described (3), should be considered in patients not only with suspected neurosyphilis but also in patients with syphilis infection and indications of a demyelinating event.

This study has limitations. Firstly, serum samples before disease onset to assess baseline MOG-IgG and sNfL were not available. Furthermore, for the first case, MOG-IgG was measured after IVIG administration, which may have resulted in a false-positive MOG-IgG result. Yet, this is unlikely to explain the positive results, especially at later follow-ups. Additionally, while we observed B cell reactivity against antigens specific for syphilis, the shared infection across the two patients, we cannot exclude that the other coexisting infections (e.g., HIV and HBV) may have also played a role in the underlying pathogenesis. Lastly, although our study provides evidence for a potential link between syphilis infection and the development of autoreactive MOG-specific B cells, cross-reactivity of patient-derived antibodies to MOG and *T. pallidum* remains to be validated molecularly.

## Data Availability

The original contributions presented in the study are included in the article/supplementary material. Further inquiries can be directed to the corresponding author.

## References

[1] Marignier R Hacohen Y Cobo-Calvo A Pröbstel AK Aktas O Alexopoulos H. Myelin-oligodendrocyte glycoprotein antibody-associated disease. Lancet Neurol. (2021) 20:762–72. doi: 10.1016/S1474-4422(21)00218-0 34418402

[2] Banwell B Bennett JL Marignier R Kim HJ Brilot F Flanagan EP. Diagnosis of myelin oligodendrocyte glycoprotein antibody-associated disease: International MOGAD Panel proposed criteria. Lancet Neurol. (2023) 22:268–82. doi: 10.1016/S1474-4422(22)00431-8 36706773

[3] Ayroza Galvão Ribeiro Gomes AB Kulsvehagen L Lipps P Cagol A Cerdá-Fuertes N Neziraj T. Immunoglobulin A antibodies against myelin oligodendrocyte glycoprotein in a subgroup of patients with central nervous system demyelination. JAMA Neurol. (2023) 80:989–95. doi: 10.1001/jamaneurol.2023.2523 PMC10407763 37548987

[4] Zimmermann M Rose N Lindner JM Kim H Gonçalves AR Callegari I. Antigen extraction and B cell activation enable identification of rare membrane antigen specific human B cells. Front Immunol. (2019) 10:829. doi: 10.3389/fimmu.2019.00829 31040853 PMC6477023

[5] Jarius S Ruprecht K Kleiter I Borisow N Asgari N Pitarokoili K. MOG-IgG in NMO and related disorders: A multicenter study of 50 patients. Part 2: Epidemiology, clinical presentation, radiological and laboratory features, treatment responses, and long-term outcome. J Neuroinflamm. (2016) 13:280. doi: 10.1186/s12974-016-0718-0 PMC5086042 27793206

[6] Ismail II Salama S. Association of CNS demyelination and COVID-19 infection: an updated systematic review. J Neurol. (2022) 269:541–76. doi: 10.1007/s00415-021-10752-x 34386902 PMC8359762

[7] Neri VC Xavier MF Barros PO Bento CM Marignier R Alvarenga RP. Case report: Acute transverse myelitis after Zika virus infection. Am J Trop Med Hygiene. (2018) 99:1419–21. doi: 10.4269/ajtmh.17-0938 PMC6283478 30277201

[8] Mbonde AA Arca KN Grill MF. Anti-MOG antibody associated encephalomyelitis in an HIV-infected patient. Mult Scler Relat Disord. (2021) 49:102753. doi: 10.1016/j.msard.2021.102753 33497849

[9] Nakamura M Iwasaki Y Takahashi T Kaneko K Nakashima I Kunieda T. A case of MOG antibody-positive bilateral optic neuritis and meningoganglionitis following a genital herpes simplex virus infection. Mult Scler Relat Disord. (2017) 17:148–50. doi: 10.1016/j.msard.2017.07.023 29055448

[10] Vieira JP Sequeira J Brito MJ. Postinfectious anti-myelin oligodendrocyte glycoprotein antibody positive optic neuritis and myelitis. J Child Neurol. (2017) 32:996–9. doi: 10.1177/0883073817724927 28820014

[11] Nakamura Y Nakajima H Tani H Hosokawa T Ishida S Kimura F. Anti-MOG antibody-positive ADEM following infectious mononucleosis due to a primary EBV infection: A case report. BMC Neurol. (2017) 17:6–9. doi: 10.1186/s12883-017-0858-6 28420330 PMC5395865

[12] Miraclin TA Sivadasan A Nair AA Rajakumar P Nair AV Joseph T. Drug resistant tuberculosis, Myelitis and MOG antibody. Neuroimmunology Rep. (2022) 2:100123. doi: 10.1016/j.nerep.2022.100123

[13] Gudenkauf JC Gadani SP McArthur JC Sotirchos ES. Neurosyphilis, MOGAD or both? A case of meningoencephalomyelitis associated with MOG-IgG seropositivity in a patient with syphilis. Neuroimmunology Rep. (2021) 1:100003. doi: 10.1016/j.nerep.2021.100003

[14] Jeyakumar N Waller S Mahant N Ramanathan S. Henderson APD. A case of CSF anti-MOG antibody-positive papillitis with intermediate uveitis in the setting of acute neurosyphilis. Neuroimmunology Rep. (2021) 1:100033. doi: 10.1016/j.nerep.2021.100033

[15] Shi M Luo D Li Z Li M Jin S Yang D. A case report of neurosyphilis coexisting with a positive MOG antibody manifested as optic neuritis. Front Neurol. (2023) 14:1258043. doi: 10.3389/fneur.2023.1258043 37859651 PMC10583717

[16] Apinyawasisuk S Poonyathalang A Preechawat P Vanikieti K. Syphilitic optic neuropathy: re-emerging cases over a 2-year period. Neuro-Ophthalmology. (2016) 40:69–73. doi: 10.3109/01658107.2015.1134586 27928388 PMC5123058

[17] Bogdanos DP Smith H Ma Y Baum H Mieli-Vergani G Vergani D. A study of molecular mimicry and immunological cross-reactivity between hepatitis B surface antigen and myelin mimics. Clin Dev Immunol. (2005) 12:217–24. doi: 10.1080/17402520500285247 PMC2275415 16295528

[18] Hirsiger JR Fuchs PS Häusermann P Müller-Durovic B Daikeler T Recher M. Syphilis reactivates latent Epstein-Barr virus reservoir via toll-like receptor 2 and B-cell receptor activation. Open Forum Infect Dis. (2019) 6:1–6. doi: 10.1093/ofid/ofz317 PMC6736073 31660400

